# Phenotypic and genotypic antimicrobial resistance profiles of clinical *Clostridioides difficile* isolates collected from private and public health settings in South Africa

**DOI:** 10.1007/s10096-025-05205-6

**Published:** 2025-07-10

**Authors:** Hlambani Shirinda, Anthony M. Smith, Mohamed Said, Ben Prinsloo, Mishalan Moodley, Marleen M. Kock, Marthie M. Ehlers

**Affiliations:** 1 Department of Medical Microbiology, School of Medicine, University of Pretoria, Pathology Building, Prinshof Campus South, University of Pretoria/NHLS, 6 Bophelo Rd, Pretoria, 0084 South Africa; 2 Lancet Laboratories, Arcadia, Pretoria South Africa; 3 National Health Laboratory Service (NHLS), Academic Division, Pretoria, South Africa; 4 Centre for Enteric Diseases, National Institute for Communicable Diseases, Johannesburg, South Africa; 5 Sequencing Core Facility, National Institute for Communicable Diseases, Johannesburg, South Africa

**Keywords:** *Clostridioides difficile*, Antimicrobial resistance, Whole genome sequencing, Lower and middle-income countries (LMICs), South Africa

## Abstract

**Purpose:**

Antimicrobial resistance (AMR) is important in the pathogenesis and spread of *Clostridioides difficile* infection (CDI). However, very little is known about the association of AMR and *C. difficile* in South Africa. The present study aimed to investigate the phenotypic and genotypic antimicrobial profiles of clinical *C. difficile* isolates.

**Methods:**

Phenotypic antimicrobial susceptibility testing (AST) and whole genome sequencing (WGS) were used to characterize the isolates.

**Results:**

The AST showed that 43 isolates were susceptible to metronidazole and vancomycin. The WGS revealed a *PnimB* promoter mutation associated with reduced metronidazole susceptibility in all sequence type (ST) 1 strains (42%, 18/43). No vancomycin resistance determinants were found. Distinct lineages displayed specific resistance traits, such as fluoroquinolone resistance in ST1 strains due to a DNA gyrase (*gyrA*) mutation (*Thr82Ile*) and multidrug resistance (MDR) in ST37 strains, which contained resistance determinants for five antimicrobial classes. Overall, 95% (40/43) of strains had AMR determinants for at least three antimicrobial classes, indicating MDR. A *qacG* efflux pump gene, conferring resistance to disinfectants, was found in all strains.

**Conclusion:**

Antimicrobial resistance to metronidazole and vancomycin remains rare, however, high MDR prevalence particularly among ST1 and ST37 strains, suggests a risk of AMR gene transmission to other pathogens. The high rate of MDR *C. difficile* may reflect extensive antimicrobial use driven by comorbidities in immunocompromised individuals, contributing to AMR. These findings underscore the importance of ongoing AMR surveillance, effective antimicrobial stewardship and infection control to manage CDI and mitigate AMR spread.

## Introduction

Antimicrobial resistance (AMR) poses a significant global health challenge requiring ongoing surveillance. However, inadequate or absent surveillance in many low- and middle-income countries (LMICs) hinders containment efforts [1]. The careless overuse of antimicrobials in various settings such as clinical treatment, agriculture, animal healthcare and food production is a primary driver of the AMR crisis [2]. If preventative measures are not implemented, AMR is projected to become the leading cause of death worldwide by 2050 [2]. The widespread use of antimicrobials, particularly clindamycin, cephalosporin and fluoroquinolones among immunocompromised individuals, has contributed to outbreaks of *Clostridioides difficile* (*C. difficile*) infections (CDI), especially given the high prevalence of HIV and tuberculosis in South Africa [3].

The success of several epidemic *C. difficile* strains is believed to be associated with an AMR phenotype, granting these strains a survival advantage in the presence of antimicrobials [4, 5]. Resistance to different antimicrobial classes has been linked to specific *C. difficile* lineages: ST1 (clade 2) shows fluoroquinolone resistance, ST11 (clade 5) demonstrates tetracycline resistance and ST37 (clade 4) exhibits multidrug resistance (MDR) [5].

In South Africa, CDI treatment predominantly relies on two antimicrobials: fidaxomicin and vancomycin [6, 7]. Despite high recurrence rates after metronidazole treatment observed in other regions, it remains the first-line option due to its affordability and availability, especially for non-severe cases [6, 7]. Fidaxomicin and vancomycin are recommended for severe CDI cases and those at risk of recurrent CDI (rCDI) [6–8]. While resistance to the primary antimicrobials used for CDI (fidaxomicin and metronidazole) remains rare, AMR significantly impacts CDI pathogenesis and spread, enabling *C. difficile* in surviving antimicrobial exposure and facilitates the emergence of resistant strains [5].

The rise of AMR in *C. difficile* is linked to the acquisition of resistance genes from other gut pathogens or environmental sources such as animals. This includes genomic mutations that alter antimicrobial target sites and changes in the expression of genes found on mobile genetic elements (MGEs). Key AMR determinants, such as those conferring fluoroquinolone resistance [e.g., mutations in DNA gyrase subunit A/B genes (*gyrA/B*)], tetracycline resistance (e.g., tetracycline resistance gene M (*tetM*)] and macrolide-lincosamide-streptogramin B (MLS_B_) resistance [e.g., erythromycin ribosomal methylase B gene (*ermB*)], play a critical role in the AMR and pathogenesis of *C. difficile* [5]. The use of these antimicrobials for treating comorbidities related to HIV, TB and post-surgical treatments disrupts intestinal microflora, facilitating the growth of *C. difficile* and its toxin production [6].

While antimicrobial testing for phenotypic and genetic resistance markers is essential for detecting and tracking AMR transmission, such testing is often not performed routinely as the condition is diagnosed mainly through non-culture methods. In South Africa, toxigenic *C. difficile* is identified in clinical stool specimens using the GeneXpert real-time PCR assay (Cepheid, USA) or a combination of enzyme immunoassays [(e.g., TECHLAB C. diff Quick Check Complete assay (TECHLAB, USA)] with GeneXpert real-time PCR (Cepheid, USA). This study aimed to investigate the phenotypic and genotypic antimicrobial resistance profiles of *C. difficile* isolates collected in South Africa.

## Methods

### Ethical approval

Ethics clearance (number 612/2022) was obtained from the Faculty of Health Science Research Ethics Committee, University of Pretoria. Permission letters were obtained from private and public diagnostic laboratories to collect residual stool specimens.

### Study settings, sample collection and isolation

*Clostridioides difficile* isolates (*n* = 43) were obtained from a study by Shirinda et al. [10] who studied *C. difficile* collected a private and public laboratory in Gauteng, South Africa. This included 22 isolates from private and 21 isolates from public laboratories.

### Phenotypic antimicrobial susceptibility testing

Antimicrobial susceptibility testing (AST) was performed on 43 *C*. *difficile* isolates using E– test (bioMérieux, France) and Fastidious Anaerobe Agar (FAA) (ThermoFischer, RSA). Briefly, the isolates were tested for susceptibility against metronidazole and vancomycin. *Clostridioides difficile* isolates were sub-cultured on 5% Columbia Horse Blood Agar (CHBA) (Thermo Scientific, RSA) and incubated (Vacutec, UK) in Mitsubishi™ AnaeroPack (Davies Diagnostics, RSA) anaerobic jar containing AnaeroPack- Anaero (Davies Diagnostics, RSA) gas-generating sachets at 37 °C for 48 h. The colonies were picked to make a 1.0 McFarland standard turbidity. The inoculum was spread on FAA (ThermoFischer, RSA) and E– test (bioMérieux, France) was applied to the surface of FAA (ThermoFischer, RSA) according to the manufacturer’s instructions. The plates were incubated (Vacutec, UK) in a Mitsubishi™ AnaeroPack (Davies Diagnostics, South Africa) anaerobic jar containing AnaeroPack-Anaero (Davies Diagnostics, RSA) gas-generating sachets at 37 °C for 48 h. Results were recorded as susceptible (S) or resistant (R) based on the break points in the EUCAST 2024 guidelines [9].

### Clostridioides difficile whole genome sequencing and analysis

The total genomic DNA of the *C*. *difficile* isolates were extracted using the Quick DNA Fungal/Bacterial Miniprep Kit (Zymogen Fermentas, USA) according to the manufacturer’s instructions. Forty-three (toxigenic and non-toxigenic) *C. difficile* DNA extracts were submitted to the Sequencing Core Facility (SCF), NICD, for WGS. The Nextera DNA Flex library prep kit (Illumina, San Diego, CA, USA) was used for library preparation, with the inclusion of a gBlock Gene Fragment (Integrated DNA Technologies, Coralville, IA, USA) as a quality control measure. The Illumina NextSeq 2000 platform (Illumina, USA) was used for sequencing at 137 100× coverage, using 2 × 150 base pairs (bp) paired-end sequencing for each flow cell.

### Bioinformatics analysis

Genomic antimicrobial resistance (resistome) determinants were characterized using a combination of tools including the Comprehensive Antibiotic Resistance Database (CARD) (version 3.2.222), ResFinder (version 4.5.0) (http://genepi.food.dtu.dk/resfinder), AMRFinderPlus and EnteroBase tools. Multidrug-resistant (MDR) isolates were defined as possessing three or more AMR genes.

### Genome sequence data availability

All sequencing data were uploaded to the public EnteroBase platform (https://enterobase.warwick.ac.uk/species/index/clostridium) and are freely available at the EnteroBase platform. In addition, sequencing data were deposited in the National Center for Biotechnology Information (NCBI) under the project accession number PRJNA1138394.

### Statistical analysis

An isolate was recorded as genotypically resistant to an antimicrobial if at least one gene or mutation known to be correlated with resistance in the genome was identified. The isolate was recorded as genotypically sensitive if no such gene or mutation was present. An isolate was recorded as phenotypically resistant to an antimicrobial if it was resistant based on the phenotypic AMR analysis.

## Results

### Phenotypic antimicrobial susceptibility and distribution of strains amongst the 43 strains

All strains (*n* = 43) were phenotypically susceptible to metronidazole and vancomycin with MIC ≤ 2 µg/mL. Metronidazole MICs ranged from 0.032 µg/mL to 0.75 µg/mL, while vancomycin MICs ranged from 0.19 µg/mL to 0.75 µg/mL. The *C. difficile* isolates included in this study were distributed into five clades and 18 distinct STs as follows: clades 1 (ST43, ST54, ST63, ST104, ST397, ST558), clade 2 (ST1), clade 3 (ST5, ST23), clade 4 (ST37), clade 5 (ST11) and two strains belonged to a novel clade (ST122 and ST558). A total of 95% (41/43) of the strains were toxigenic and 5% (2/43) non-toxigenic.

### Genotypic antimicrobial resistance to metronidazole andvancomycin

The WGS of the strains showed that the most clinically relevant AMR determinant was the mutation in the promoter region of the 5-nitroimidazole resistance gene B (P*nimB*^*G*^), associated with metronidazole resistance, this mutation was detected in 42% (18/43) of strains, 83% (15/18) were from public and 17% (3/18) were from private health settings [11]. All strains that carried P*nimB*^*G*^ belong to ST1. No pCD-METRO plasmid was identified in any of the strains. None of the strains harboured key AMR determinants (*murG* mutations) associated with vancomycin resistance.

### Resistance to other important antimicrobials

The intrinsic genes for beta-lactamase, *CDD-1* and *CDD-2*, conferring resistance to cephalosporins were detected in all the genomes (*n* = 43). An amino acid mutation (*Thr82Ile*) in the DNA gyrase A (*gyrA*), associated with fluoroquinolone resistance was identified in 44% (19/43) of the genomes, including 95% (18/19) of the ST1 strains and one ST37 strain. The *C. difficille* efflux system protein A (*cdeA*), associated with fluoroquinolone resistance was identified in three genomes, including two ST122 strains and one ST54 strain. Efflux pump genes, the macrolide efflux protein H gene (*mefH*) and the macrolide-streptogramin B resistance gene *msr (D)*, were each identified in four strains, specifically in ST63 (*n* = 1), ST122 (*n* = 2) and ST558 (*n* = 1). Both efflux pump genes are linked to macrolide resistance. Additionally, the *qacG* gene, an efflux pump gene associated with resistance to disinfecting agents and antiseptics was identified in all the genomes (*n* = 43).

A total of 65% (28/43) of the genomes analysed contained at least one MGE such as mobilizable transposons (Tn), unit transposons (*Tn6218*), conjugative transposons (CTn) and insertion sequence (IS) (Table 1). In total, 11 distinct MGEs were identified across these genomes. The most frequently detected transposons were: *Tn610*5 [64%, 18/28] and *CTn7* [25%, 7/28]. Notably, 82% (23/28) of the genomes with MGEs also carried AMR genes (*n* = 11). The analysis revealed that the most associated genes within MGEs were aminoglycoside O-phosphotransferase [*aac (6’)-Ie-aph (2’’)-Ia*], erythromycin (and clindamycin and streptogramin B) ribosomal methylase B gene (*ermB*) and tetracycline resistance protein M (*tetM*) (Table 2). The *Tn6105* was particularly linked to aminoglycoside resistance, as it consistently harboured the *aac (6’)-Ie-aph (2’’)-Ia* gene and was associated with all ST1 genomes. An association between *Tn6009* and *Tn6194* was also observed in both ST37 strains harbouring the *ermB* and *tetM* genes. Additionally, the ST54 strain, contained the highest number of MGEs [64%, 7/11], including *Tn6218*,* Tn5801(B6)*,* CTn1*,* CTn4*,* CTn5*,* CTn6 and CTn7*. Interestingly, ST122, belonging to an unknown clade, harboured the highest number of acquired AMR genes and efflux pumps (*n* = 5), despite the absence of any identified transposon. This suggests that this strain may employ an alternative mechanism for AMR. Table 2 displays the frequency of MGEs across 28 genomes and the corresponding sequence types, Table 3 displays the proportion of AMR genes harboured in MGEs in the *C. difficile* genomes and Table 1 shows the AMR genes harboured in MGEs.

**Table 1:** Antimicrobial-resistance genes harboured in each mobile genetic elements

Mobile genetic element	Number of genomes in which antimicrobial resistance gene was detected *n* = 28 (%)						
	aac (6’)-Ie-aph (2’’)-Ia	tetA	tetB	tetM	ermB	ant (6)-Ia	catP
*Tn6105*	18(64%)	-	-	-	-	-	-
*Tn6218*	1 (4%)	1 (4%)	1 (4%)		2 (7%)	-	-
*Tn6194*	-	-	-	2 (7%)	2 (7%)	1 (4%)	
*Tn4453*	1 (4%)	-	-		-	1 (4%)	1 (4%)
*Tn6009*	-	-	-	2 (7%)	2 (7%)	1 (4%)	-
*Tn5801(B6)*	1 (4%)	-	-	1 (4%)	1 (4%)	-	-
*CTn1*	1 (4%)	-	-	1 (4%)	1 (4%)	-	-
*CTn2*	1 (4%)	-	-	1 (4%)	1 (4%)	-	-
*CTn4*	1 (4%)	-	-	1 (4%)	1 (4%)	-	-
*CTn5*	1 (4%)	-	-	1 (4%)	1 (4%)	-	-
*CTn6*	1 (4%)	-	-	1 (4%)	1 (4%)	-	-
*CTn7*	1 (4%)	-	-	1 (4%)	1 (4%)	1 (4%)	1 (4%)
*ISRob1*	1 (4%)	-	-	-	-	1 (4%)	1 (4%)

**Table 2:** Frequency of mobile genetic elements across 28 genomes and the corresponding sequence types from the current study

Mobile Genetic Element	Corresponding Sequence types (*n*)	Genomes in Which MGEs Were Identified (*n*) (%)	Genomes with MGEs in Which at Least One AMR Gene Was Identified (*n*) (%)
*Tn6105*	ST1 (18)	18 (64%)	18 (64%)
*Tn6218*	ST3 (1), ST54 (1)	2 (7%)	2 (7%)
*Tn6194*	ST37 (37)	2 (7%)	2 (7%)
*Tn4453*	ST35 (1)	1 (4%)	1 (4%)
*Tn6009*	ST37 (2)	2 (7%)	2 (7%)
*Tn5801(B6)*	ST54 (1)	1 (4%)	1 (4%)
*CTn1*	ST54 (1)	1 (4%)	1 (4%)
*CTn2*	ST54 (1)	1 (4%)	1 (4%)
*CTn4*	ST54 (1)	1 (4%)	1 (4%)
*CTn5*	ST54 (1)	1 (4%)	1 (4%)
*CTn6*	ST54 (1)	1 (4%)	1 (4%)
*CTn7*	ST2 (1), ST3 (2), ST23 (1), ST29 (1), ST35 (1), ST54 (1)	7 (25%)	2 (7%)
*ISRob1*	ST23 (1)	2 (7%)	1 (4%)
*IS256*	ST35 (1), ST37 (2)	1 (4%)	1 (4%)

**Table 3:** The proportion of antimicrobial-resistance genes harboured in mobile genetic elements in the *Clostridioides difficile* genomes

Antimicrobial-resistance determinant	Number of isolates with acquired antimicrobial resistant determinant *n* = 28 (%)
*aac (6’)-Ie-aph (2’’)-Ia*	21 (75%)
*tetA (P)*	5 (18%)
*tetB (P)*	5 (18%)
*tetM*	4 (14%)
*ermB*	4 (14%)
*ermG*	4 (14%)
*msr* (*D*)	4 (14%)
*mef* (*A*)	4 (14%)
*ermT*	2 (7%)
*ant (6)-Ia*	2 (7%)
*catP*	1 (4%)

## Discussion

Phenotypic resistance to either metronidazole or vancomycin antimicrobials was not observed in the current study. The current study’s results were similar to those conducted in a tuberculosis hospital in Cape Town, South Africa, where none of the *C. difficile* strains were resistant to metronidazole and vancomycin [12]. A study in Israel reported similar results, where all isolates were susceptible to both metronidazole and vancomycin [13].

The results are similar to studies conducted in Brazil, Ireland and Thailand where all the strains were susceptible to metronidazole and vancomycin [14, 15, 17]. Another study in Australia reported susceptibility to metronidazole while only 5% were resistant to vancomycin [18]. Fidaxomicin, one of the recommended antibiotics for CDI with a narrow spectrum of activity, was not included in the current study because we could not source the fidaxomicin for laboratory testing in South Africa and could not shipped from international suppliers.

A total of 43 strains representing 18 STs were characterised. Except for two strains belonging to ST11 and ST29, all 41 strains harboured at least one resistance determinants in three or more antimicrobial classes and were therefore classified as MDR. There was an agreement between phenotypic and genotypic results as AMR determinants (pCD-METRO plasmid and *murG* mutations) known for causing resistance to metronidazole and vancomycin were not identified. However, a chromosomal mutation (*PnimB*^*G*^ promoter mutation) associated with reduced susceptibility to metronidazole was identified. Additionally, no AMR determinant associated with fidaxomicin resistance was identified using WGS.

The success of several epidemic *C. difficile* strains is believed to be linked to an AMR phenotype, which provides these strains with a survival advantage in the presence of antimicrobials [5]. Resistance to various antimicrobial classes has been associated with specific *C. difficile* lineages: fluoroquinolone resistance with ST1 (clade 2) and MDR with ST37 (clade 4) [5]. The current study provided genotypic evidence to support these associations. This study detected resistance to these antimicrobial classes through various AMR mechanisms, including chromosomal resistance genes, mutations, efflux pumps and MGEs such as plasmids, mobilizable transposons, conjugative transposons, unit transposons and insertion sequences.

The resistance mechanism of *C. difficile* to fluoroquinolones is usually caused by alterations of targeted structures (*gyrA* and/or *gyrB*) through nucleotide substitutions such as *Thr82IIe* in *gyrA* [5, 14]. The *gyrA* (*Thr82IIe*) mutations were present in all ST1 strains and one ST37 strain. None of the strains showed *gyrB* gene mutations. Similarly, Imwattana et al. [5] found that most AMR was conferred by point substitutions solely within the *gyrA* subunit. According to the literature, fluoroquinolone resistance plays a significant role in the spread of hypervirulent strain *C. difficile* ST1 which caused substantial outbreaks across North America and Europe in the 2000s [15]. The ST37 strains were MDR harbouring AMR determinants to five antimicrobial classes including nitroimidazole, glycopeptide, macrolides, fluoroquinolones, carbapenems and tetracycline. There was an association between ST1 and specific AMR determinants, *PnimB*^*G*^ promoter mutation, in *gyrA* mutation (*Thr82IIe*) and *Tn6105* mobilizable transposon which harboured *(6′)-Ie-aph* (*2″*)*-Ia* as all the ST1 strains carried these determinants. The *Tn6194* contained excisionase (*xis*) and integrase (*int*) genes and can integrate at different sites in the chromosome [5, 15]. The *Tn6194* was the most common *ermB-*containing element in European Clinical *C. difficille* isolates [14, 15]. In the current study, *Tn6194* was identified in two ST37 strains which also contained the *ermB* gene. The *Tn6218* unit transposon contained *int* and *xis* genes and was located on the chromosome [5, 15]. In the current study, *Tn6218* was identified in ST3 and ST54 strains which also carried *ermB* genes. The *Tn4453* mobilizable transposon was identified in one ST35 strain which also carried chloramphenicol resistant gene (*catP*). These findings were in agreement with the global trends [5, 14, 15].

The novel ST122 (same toxigenic profile as ST1 as it carries binary toxin) harboured resistant genes belonging to six (nitroimidazole, glycopeptide, macrolides, fluoroquinolones, beta-lactam and tetracycline) antimicrobial classes. This indicates the importance of not overlooking this strain when analysing data despite that it is not allocated to any clade as the AMR determinants within the strain can be disseminated to other pathogens. Sequence type 11 and ST668 strains had low number of genomic resistance determinants.

Although *C. difficile* is intrinsically resistant to aminoglycosides, some strains contained genes that confer resistance to aminoglycosides and can spread to other bacteria, this included genes such as *aac (6′)-Ie-aph* (*2″*)*-Ia.* In the current study, the *aac (6′)-Ie-aph (2″)-Ia* gene was identified in 49% (21/43) of the strains, including ST1 (*n* = 18) and one strain each of ST3, ST35 and ST54. All ST1 strains acquired the *aac (6′)-Ie-aph (2″)-Ia* gene chromosomally via the Tn6105 mobilizable transposon, while ST3 and ST54 acquired the gene chromosomally through the *Tn6218* unit transposon. It has been reported that anaerobes are intrinsically resistant to aminoglycosides, with high MICs reported (MIC_50_ 120 mg/L, 95% CI 62–250; MIC90 200 mg/L 95% CI 78–490) [16].

Although the presence of such genes should not necessarily be correlated with aminoglycoside resistance in *C. difficile*, their contribution to aminoglycoside resistance through being spread to other species such as *Enterococcus faecium*, *Staphylococcus aureus*, *Klebsiella pneumoniae*, *Acinetobacter baumannii*, *Pseudomonas aeruginosa* and *Enterobacter* species (ESKAPE) is important [15].

The key strengths of the current study include the successful application of online bioinformatics tools such as EnteroBase, CARD and ResFinder for AMR profiles without requiring advanced bioinformatics skills. This made the genomic analysis process more accessible and efficient. The key limitation was the inability to phenotypically test susceptibility to other antimicrobials due to financial constraints. Another limitation was geographic and healthcare setting limitations: because the study was confined to only 43 clinical isolated from stool specimens collected from one private and one public laboratory. This may not fully represent the broader epidemiology of *C. difficile* in the entire country, limiting the generalizability of the finding. These limitations highlight the need for a more expansive, multi-center, interdisciplinary studies with comprehensive testing of antimicrobials.

In conclusion, all isolates were susceptible to metronidazole and vancomycin. The detection of AMR genes linked to antimicrobials associated with *C. difficile* pathogenesis may reflect the widespread use of these drugs among immunocompromised individuals, driven by the high prevalence of HIV and tuberculosis in South Africa [17]. Antimicrobial determinants associated with macrolides, MLS_B_, cephalosporins, fluoroquinolones, aminoglycosides, tetracyclines and chloramphenicol were identified in the current study. Although only two antimicrobials (fidaxomicin and vancomycin) are recommended for the treatment of CDI in South Africa, future studies should consider including other antimicrobials on phenotypic testing to investigate the correlation between phenotyping and genotypic AMR of the antimicrobials associated with *C. difficile* pathogenesis. This study identified key AMR that can potentially lead to outbreaks of CDI and include fluoroquinolones, MLS_B_ and tetracyclines. This provides an opportunity to develop a focused antimicrobial stewardship policy, targeting specific antimicrobial classes based on the prevalent *C. difficile* strains in the region. The findings from the current study contributed to the knowledge of AMR and resistance mechanisms of *C. difficile*. Additionally, the detection of disinfectant- and antiseptic-resistant efflux pumps further underscores the importance of reinforcing infection control strategies.

## Data Availability

No datasets were generated or analysed during the current study.
